# Size-Dependence and High Temperature Stability of Radial Vortex Magnetic Textures Imprinted by Superconductor Stray Fields

**DOI:** 10.1021/acsami.3c17671

**Published:** 2024-04-02

**Authors:** David Sanchez-Manzano, Gloria Orfila, Anke Sander, Lourdes Marcano, Fernando Gallego, Mohamad-Assaad Mawass, Francesco Grilli, Ashima Arora, Andrea Peralta, Fabian A. Cuellar, Jose A. Fernandez-Roldan, Nicolas Reyren, Florian Kronast, Carlos Leon, Alberto Rivera-Calzada, Javier E. Villegas, Jacobo Santamaria, Sergio Valencia

**Affiliations:** † Laboratoire Albert Fert, CNRS, Thales, Université Paris-Saclay, 91767 Palaiseau, France; ‡ GFMC. Department Física de Materiales. Facultad de Física. Universidad Complutense. 28040 Madrid, Spain; § Helmholtz-Zentrum Berlin, Albert-Einstein Str. 15, 12489 Berlin, Germany; ∥ Department of Physics, Faculty of Science, University of Oviedo, 33007 Oviedo, Spain; ⊥ Center for Cooperative Research in Biomaterials (CIC biomaGUNE), Basque Research and Technology Alliance (BRTA), Paseo de Miramón 194, 20014 Donostia-San Sebastián, Spain; # Institute for Technical Physics Karlsruhe Institute of Technology, 76344 Eggenstein-Leopoldshafen, Germany; ¶ Helmholtz-Zentrum Dresden-Rossendorf e.V., Institute of Ion Beam Physics and Materials Research, 01328 Dresden, Germany

**Keywords:** superconductor, ferromagnet, XMCD-PEEM, magnetic imprint, vortex, magnetic texture

## Abstract

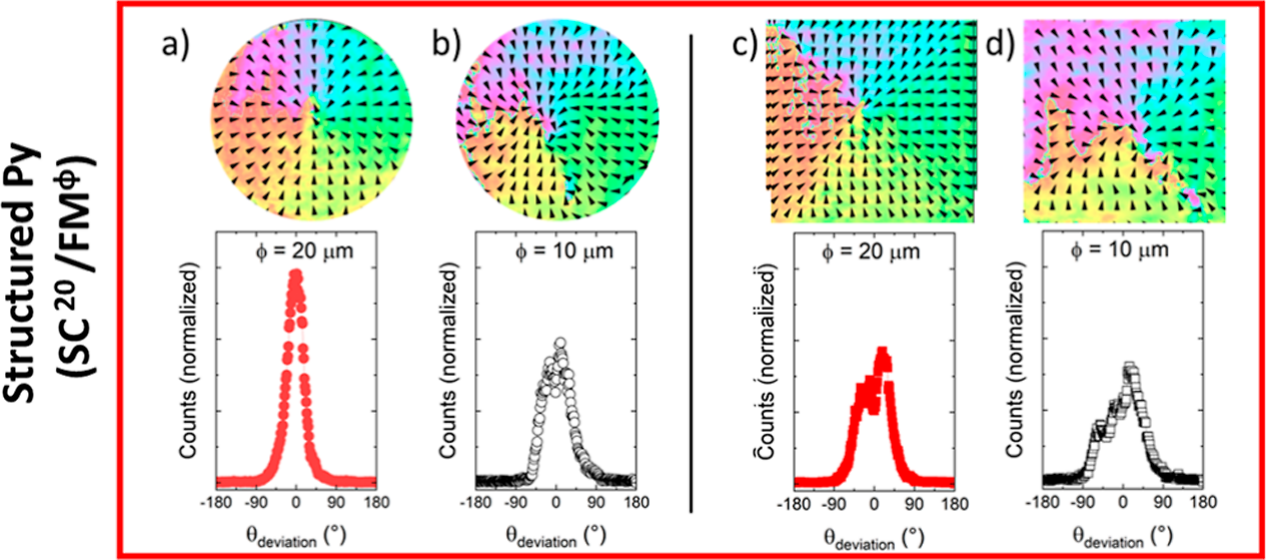

Swirling spin textures, including topologically nontrivial states, such as skyrmions, chiral domain walls, and magnetic vortices, have garnered significant attention within the scientific community due to their appeal from both fundamental and applied points of view. However, their creation, controlled manipulation, and stability are typically constrained to certain systems with specific crystallographic symmetries, bulk or interface interactions, and/or a precise stacking sequence of materials. Recently, a new approach has shown potential for the imprint of magnetic radial vortices in soft ferromagnetic compounds making use of the stray field of YBa_2_Cu_3_O_7-δ_ superconducting microstructures in ferromagnet/superconductor (FM/SC) hybrids at temperatures below the superconducting transition temperature (*T*_C_). Here, we explore the lower size limit for the imprint of magnetic radial vortices in square and disc shaped structures as well as the persistence of these spin textures above *T*_C_, with magnetic domains retaining partial memory. Structures with circular geometry and with FM patterned to smaller radius than the superconductor island facilitate the imprinting of magnetic radial vortices and improve their stability above *T*_C_, in contrast to square structures where the presence of magnetic domains increases the dipolar energy. Micromagnetic modeling coupled with a SC field model reveals that the stabilization mechanism above *T*_C_ is mediated by microstructural defects. Superconducting control of swirling spin textures, and their stabilization above the superconducting transition temperature by means of defect engineering holds promising prospects for shaping superconducting spintronics based on magnetic textures.

## Introduction

The rise of spintronics has stimulated the interest in topologically nontrivial spin configurations, such as skyrmions,^1−3^ merons,^4−6^ and magnetic (radial) vortices,^7−9^ promoting the search for new materials, methods, and/or configurations in which these structures can be created, stabilized, and controlled. There have been significant advances in our understanding of the physics governing the formation of these nontrivial magnetic domain configurations. However, a method for creating and stabilizing complex spin textures, applicable to a large variety of compounds, is yet missing. Recently, a new approach based in the use of hybrid superconductor/ferromagnet (SC/FM) microstructures has demonstrated the possibility to generate and control swirling spin textures^10−17^ with some degree of stability even above the superconducting transition temperature.^11,16^

Superconductivity and ferromagnetism are two electronic ground states which, despite their antagonistic character, may become synergistic in superconductor/ferromagnet hybrids. They yield exciting responses as, for example, the recently demonstrated long-range supercurrent and Josephson effects driven by equal spin triplet superconducting correlations^18−20^ which coexist with ferromagnetism.

A wide category of effects in SC/FM hybrids involves the influence of the ferromagnet on the superconducting ground state. This influence is mediated by the stray fields of ferromagnetic domains or magnetic chiral structures (like magnetic vortices, skyrmions, or domain walls) on the dissipation properties and critical current characteristics of the superconductor.^21−27^ For instance, the presence of a magnetic vortex in the ferromagnetic barrier of a Josephson junction can tailor the supercurrent pathways making it to behave as a SQUID^28^ or 0-π SQUID.^29^

The opposite, that is, the role played by superconducting stray fields in the magnetic ground state of ferromagnets, has comparatively received less attention. To this regard, Palau et al.^17^ and Sander et al.^16^ have shown the possibility of crafting swirling spin textures in ferromagnetic systems by making use of the magnetic stray fields generated by the trapped flux in structured type II superconductors. Below the superconducting transition temperature, the application and removal of an out-of-plane magnetic field yields the generation of screening supercurrents due to the penetration, pinning, and expulsion of magnetic flux quanta.^30,31^ Within the mixed state, these supercurrents flow following the geometrical contour of the SC structure,^32−34^ with a geometry and sense of rotation which depend on magnetic history.^16,17^ Supercurrents present after removal of the external magnetic field, give rise to a stray magnetic field whose strength and direction varies locally,^35^ see Supporting Information, Section 1. Typical values of the vortex density for 100 mT (the field used in this study) yield 4.8 × 10^13^ vortices/m^2^, which corresponds to an intervortex distance (assuming a square lattice for YBCO) of 140 nm. In ferromagnetic systems with perpendicular magnetic anisotropy (PMA), the out-of-plane component of the superconductor stray-field can be employed to imprint unusual magnetic textures.^16^ The imprint is stabilized by the PMA so that it remains even when supercurrents have vanished for temperatures above *T*_C_. On the other hand, Palau et al. showed that in ferromagnetic layers with in-plane magnetic anisotropy, the in-plane components of the SC stray-field can be utilized to imprint magnetic domain distributions akin to radial vortices with a lateral size of 20 μm.^17^ In these, the in-plane magnetization can point toward or away from the core along radial directions orthogonal to the contour of the superconducting microstructure. This type of magnetization distribution is not energetically favored due to large dipolar energies.^36^ While millimeter size structures are expected to retain at *T* > *T*_C_ some memory of the imprinted state,^11^ for smaller structures, the disappearance of the SC stray-field above *T*_C_ is expected to lead to its relaxation to an energetically more favorable magnetic state, such as a conventional vortex or a multidomain configuration.^37^

Here, we explore how the reduction of the lateral size of the SC structure affects the SC imprint of radial vortex-like magnetic spin textures on YBa_2_Cu_3_O_7−δ_/Ni_80_Fe_20_ (YBCO/Py) hybrids. Finite-difference micromagnetic modeling coupled with the YBCO field modeling indicate that the radially inhomogeneous field distribution of the superconductor enables the imprint of these topologically nontrivial magnetic domain distributions below *T*_C_ for lateral sizes down to submicrometer. Experimentally, we obtain radial vortex-like imprints down to 2 μm, most likely limited by the presence of surface defects. Interestingly, although increasing the temperature above *T*_C_ leads to the disappearance of the stabilizing SC stray field and the relaxation of the imprinted magnetic domain pattern, the remnant spin texture retains a significant memory of the imprinted state (nonvolatile). The robustness of this state and the origin of this memory effect are discussed in terms of pinning of domain walls by YBCO surface defects, which contribute to stabilize its topology.

## Experimental Section

We have fabricated microstructured SC/FM hybrids with square (⊡) and disc (⊙) shapes based on YBa_2_Cu_3_O_7−δ_/Ni_80_Fe_20_ (YBCO/Py). Permalloy was selected as a FM system due to its low coercivity, saturation field, and in-plane magnetic anisotropy which eases the imprint of magnetic domains by means of the magnetic stray fields generated by the SC. Two types of SC/FM systems have been investigated. The first system is made of samples for which a continuous Py film has been deposited on top of YBCO structures of different sizes, as in ref (17). The second type consists of samples where the Py has been structured with the same shape but with sizes smaller than that of the SC microstructure underneath (20 μm). From now on, we will use the notation (⊡, ⊙)SC^Ø^/FM^cont^ to refer to (square or circular) structures with continuous Py layer and variable SC element size and (⊡, ⊙)SC^20^/FM^Ø^ to refer to structures with structured Py on top of 20 μm SC islands, respectively. The superscript Ø indicates the lateral size of the SC or FM in μm, as well as the size of imprinted magnetic domains.

**Figure 1:**
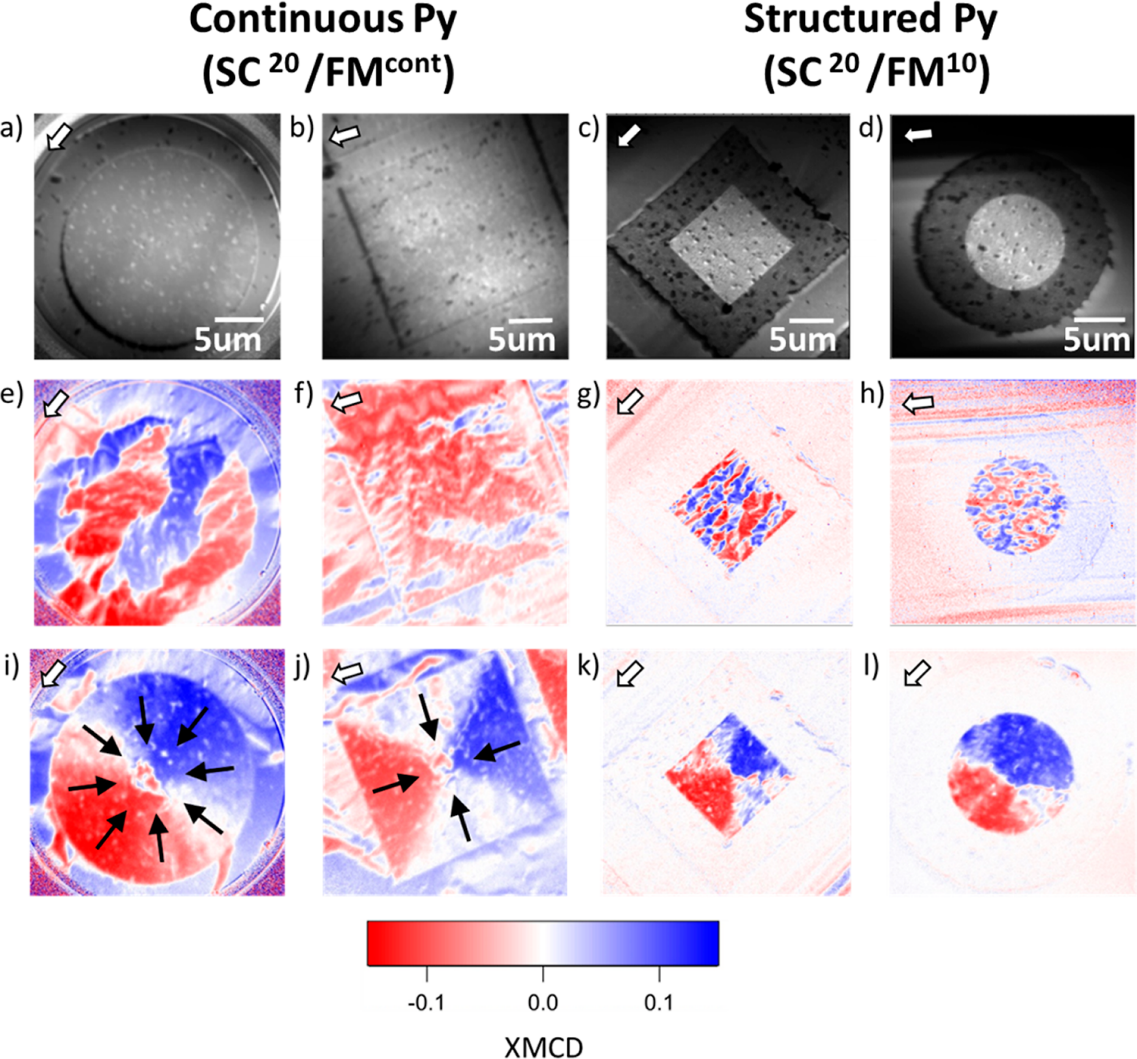
XAS PEEM images obtained at *T* = 50 K for hybrid SC/FM structures with continuous Py layer on top (a) ⊙ SC^Ø^/FM^cont^ and (b) ⊡ SC^Ø^/FM^cont^ and with structured Py (c) ⊡ SC^20^/FM^10^ and (d) ⊙ SC^20^/FM^10^. (e–h) XMCD images obtained at *T* = 50 K after a zero-field cool process. (i–l) Corresponding XMCD images after an out-of-plane magnetic field pulse of −100 mT. Black arrows indicate the direction of the imprinted magnetization which resembles that of a magnetic radial vortex. White arrows signal the in-coming X-ray beam direction.

A 250 nm thick superconducting YBCO layer was epitaxially deposited on top of (001)-oriented Nb-doped SrTiO3 substrates by means of high oxygen pressure (3.4 mbar) dc magnetron sputtering at 900 °C. Following growth, in situ annealing in pure oxygen for 30 min at 550 °C was performed to ensure an optimal oxygen stoichiometry. Growth conditions, optimized for epitaxial *c*-axis growth, lead to superconducting films with a transition temperature (89 K) close to that of the bulk (92 K). As-grown films are decorated by the presence of CuO surface precipitates characteristic of the high oxygen pressure sputtering growth and are difficult to avoid. Precipitates are randomly distributed with diameter sizes varying between ca. 100 and 500 nm, see Figure 1 and Supporting Information of ref (16). Superconducting YBCO square and disc structures, with lateral sizes (or diameter) ranging between Ø = 1 and 20 μm, were defined by means of electron beam lithography and etching. A 4 nm thick layer of permalloy (Ni_80_Fe_20_) was deposited by means of magnetron sputtering at room temperature after the structuring of YBCO. All samples were capped with 3 nm of Al to prevent oxidation. A second lithographic process (see Methods) followed in those cases where the FM was structured into the same shape as the SC.

Single out-of-plane magnetic field pulses of +100 or −100 mT were applied at *T* < *T*_C_ to induce the supercurrent distribution that generates a magnetic stray field from the SC. The imprint is enabled when the in-plane component of the stray field overcomes the coercive field (H_c_) of the Py and exceeds or falls slightly below the saturation field (H_s_). While the in-plane stray field of the SC is strongly temperature dependent^38^ below the *T*_C_ of the YBCO, both H_c_ and H_s_ have a weak dependence on temperature in the temperature range of the experiment. Following this, a temperature of 50 K was selected for the imprint (the lowest temperature achievable by the experimental system) to maximize the SC stray field.

The impact of the magnetic stray field of the SC on the magnetic domain structure of the FM was imaged by means of X-ray photoemission electron microscopy (XPEEM). X-ray magnetic circular dichroism (XMCD), measured at the Fe *L*_3_-edge (707 eV), was used as a magnetic contrast mechanism (see Methods). XMCD images as a function of T have been obtained to evaluate the impact of the disappearance of the SC stray field above *T*_C_ on the imprinted magnetic domains. All XMCD images were obtained at magnetic remanence, i.e., in the absence of external magnetic fields.

## Results

After a zero-field cool process down to 50 K (*T* < *T*_C_) hybrid SC/FM structures with continuous (Figure 1a,b) and structured (Figure 1c,d) Py present a magnetic multidomain state (Figure 1e–h). An out-of-plane magnetic field pulse of −100 mT (see Methods) triggers a profound modification of the Py magnetic domain distribution due to the self-field of the SC structure; see Figure 1j–l. The magnetization direction sensitivity of XMCD excludes the possibility that the resulting magnetic domain pattern is a magnetic vortex. The XMCD signal distribution for a conventional vortex would look alike to that depicted in Figure 1j–l but under a rotation of ±90° (Supporting Information Section S2). The resulting magnetic domain state, both for square and disc-shaped structures, features a radial vortex-like configuration where the local magnetization is orthogonal to the microstructure contour and points toward its geometrical center.^17^ Both the resolution and the in-plane magnetic sensitivity of the experimental setup prevent “visualization” of the central core predicted by micromagnetic simulations (Supporting Information, Section S3). Consequently, from now on, we restrict our analysis to the in-plane components of imprinted magnetization.

In the case of discs, the radial magnetization direction features a continuous and smooth 360° rotation around its center, similar to that reported for radial vortices.^9,36^ On the other hand, square structures display magnetic domain walls along the diagonals splitting the magnetic domain state into four equal triangular-shaped head-to-head magnetic domains, where the magnetization direction rotates 90° between adjacent ones. Full reversal of the magnetic domain structure can be achieved by changing the sign of the out-of-plane magnetic field pulse, whereas intermediate states can be obtained by changing its strength.^17^

**Figure 2:**
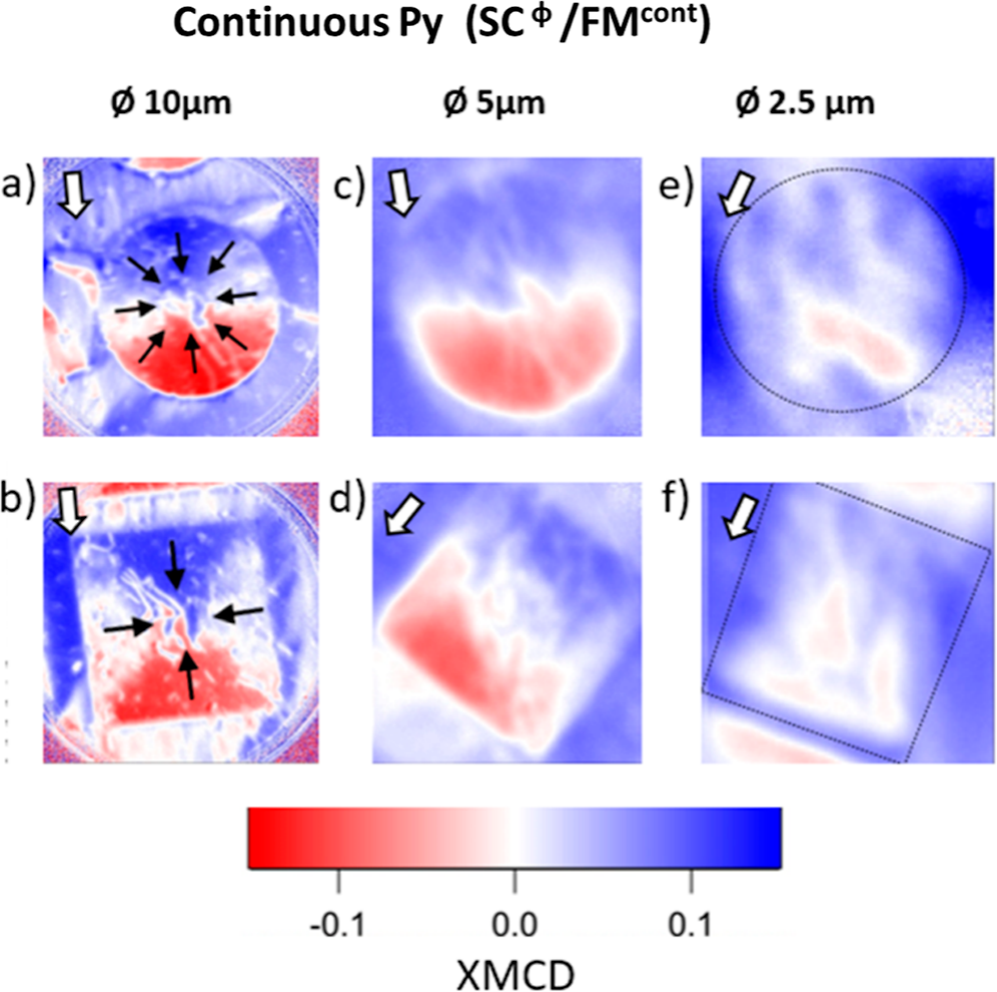
XMCD images obtained at *T* = 50 K after a magnetic field pulse of −100 mT for (a,b) (⊙, ⊡)SC^10^/FM^cont^, (c,d) (⊙, ⊡)SC^5^/FM^cont^, and (e,f) (⊙, ⊡)SC^2.5^/FM^cont^. XMCD corresponding to panels (c,f) have been averaged over 10, 7, 8, and 12 similar structures, respectively. Images corresponding to panels (a,b) correspond to a single structure. Black arrows indicate the direction of the imprinted magnetization which resembles that of a magnetic radial vortex domain. White arrows signal the in-coming X-ray beam direction.

**Figure 3:**
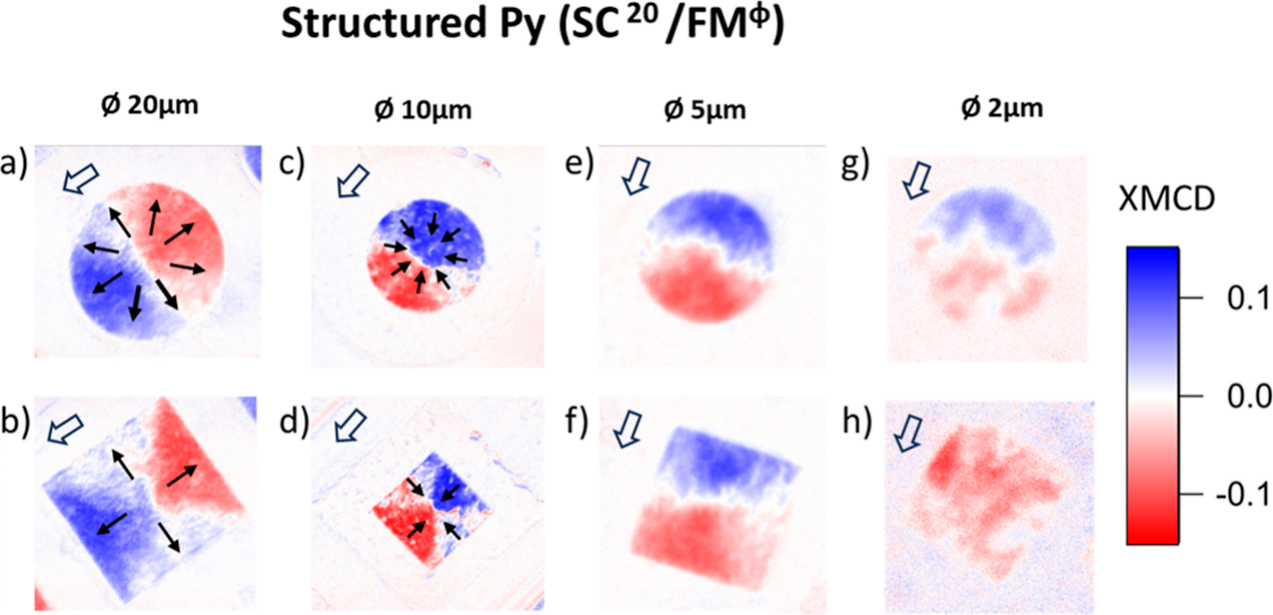
XMCD images obtained at *T* = 50 K after a magnetic field pulse of −100 mT for (a,b) (⊙, ⊡)SC^20^/FM^20^, and after a magnetic pulse of +100 mT for (c,d) (⊙, ⊡)SC^20^/FM^10^, (e,f) (⊙, ⊡)SC^20^/FM^5^, and (g,h) (⊙, ⊡)SC^20^/FM^2^ structures. XMCD corresponding to panels (a,b,e,g,h) have been averaged over 8, 10, and 4 similar structures, respectively. Images corresponding to panels (c,d) correspond to a single structure. Black arrows indicate the direction of the imprinted magnetization which resembles that of a magnetic radial vortex domain. White arrows signal the in-coming X-ray beam direction.

The effectiveness of the imprint for (⊡, ⊙)SC^Ø^/FM^cont^ and (⊡, ⊙)SC^20^/FM^Ø^ structures as a function of the imprint size Ø is shown in Figures 2 and 3, respectively. XMCD images have been averaged over several similar structures to mask nonmagnetic regions linked to surface defects. Both sample systems show a decrease in the efficiency of the imprint of radial vortex magnetization distributions for smaller Ø. Structures with a continuous Py layer, (⊡, ⊙)SC^Ø^/FM^cont^, show a steady decrease of the XMCD strength as the size is reduced. For these samples, the reduction of the lateral size of the SC element leads to an overall decrease of the stray field of the superconductor,^39,40^ see Supporting Information, Figures S1 and S2. Yet, the in-plane fields generated by (⊡, ⊙)SC^2.5^ structures are high enough to align the magnetization of a 4 nm thick Py film (Supporting Information, Section 4).

The efficiency of the imprint improves when the Py is structured on top of SC islands with the largest stray field (Ø = 20 μm), i.e., for (⊡, ⊙)SC^20^/FM^Ø^ samples, see Figure 3. Such an improvement is evidenced by the fact that XMCD averaged images corresponding to (⊡, ⊙)SC^20^/FM^20^, (⊡, ⊙)SC^20^/FM^10^, and (⊡, ⊙)SC^20^/FM^5^ show a similar XMCD signal distribution, which is expected from a deterministic imprint. It is feasible to imprint structures of a minimum size (side) of Ø = 5 μm in ⊡ square structures and Ø = 2 μm diameter for ⊙ circles.

**Figure 4:**
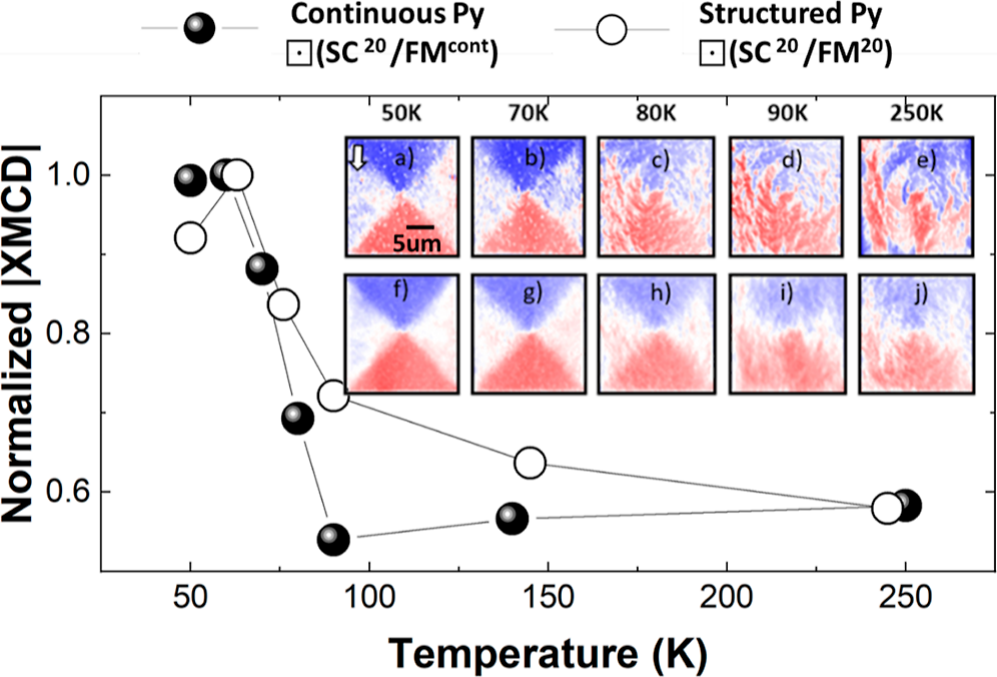
Main panel: temperature dependence of the absolute XMCD integrated intensity averaged over 10 similar ⊡ SC^20^/FM^cont^ (full dots) and ⊡ SC^20^/FM^20^ (open dots) structures. The integration area is defined over “blue” and “red” triangular domains at *T* = 50 K. The signal is normalized to its maximum. (a–e) and (f–j) XMCD images obtained as a function of temperature after a magnetic field pulse of −100 mT for ⊡ SC^20^/FM^cont^ structures. (a–e) correspond to a single structure. (f–j) are averaged over 10 similar structures. A white arrow signals the in-coming X-ray beam direction. XMCD scale as in previous figures.

The stability of the imprint as the temperature is increased above the SC transition has been investigated by obtaining XMCD images as a function of temperature after a superconducting imprint at 50 K for ⊡ SC^20^/FM^cont^ and ⊡ SC^20^/FM^20^ structures. Fine details concerning the relaxation of the imprinted magnetic domain depend on the particularities of each structure, namely, the distribution of defects. To wash out these particularities and reveal their common behavior we depict in the main panel of Figure 4 the absolute value of the XMCD signal averaged over 10 similar structures as a function of T. The signal has been integrated over areas defined by blueish (XMCD > 0) and reddish (XMCD < 0) domains at 50 K (Figure 4f). For the sake of comparison, the signal has been normalized. Similar results were obtained for structured and continuous Py. As *T* increases, the |XMCD| signal decreases, reaching about 60% of its initial value at *T* = 90 K and remaining roughly constant up to 250 K. This initial reduction of the XMCD signal is associated with the decrease and final disappearance of the SC stray-field at *T*_C_ leading to the relaxation of the imprinted magnetic state. The nonvanishing XMCD signal above *T*_C_ indicates that imprinted domains do not relax to a conventional vortex pattern configuration or multidomain state once the SC stray-field vanishes. Inset panels a–e depict XMCD images obtained for one of the ⊡ SC^20^/FM^cont^ structures. At 80 K, close to *T*_C_ (89 K), there is a partial relaxation of the imprinted magnetic domain state, as evidenced by the appearance of dendritic domains and an overall change of the XMCD strength. In between 80 and 250 K, there is little variation. Similar qualitative results were obtained for all structures measured. Average XMCD images, depicted in panels f–j, reveal a common behavior. At all temperatures, the average images feature, albeit with some relaxation at higher temperatures, the magnetic domain pattern imprinted at 50 K.

## Discussion

The imprint of radial magnetic vortex configurations in samples with a continuous Py layer is not observed for confined structures with diameters Ø ≤ 2.5 μm. This is ascribed to an interplay between the stray field generated by the superconducting layer compared to the effective anisotropy field in confined geometries, which includes the dipolar field arising from the ferromagnetic Py regions situated between the structures (Supporting Information, Section 5). Indeed, as shown in Figure 1e,f, (⊡, ⊙)SC^Ø^/FM^cont^ structures exhibit a characteristic domain length comparable to or larger than the lateral dimension of the largest imprinted Py region, which highlights the significant role played by the long-range dipolar field interactions originating from Py regions away from the SC structures. Structured Py samples have two benefits in this respect permitting a more efficient and a lower size-limit imprint. First, the SC stray field, which competes against an increase of the coercive field as FM size is reduced, is maximized as FM structures sit on top of the largest SC elements. Second, there are no FM regions in between superconducting islands, and therefore also no dipolar fields originating in these regions which could compete with the SC magnetic stray field. However, we observe no imprint for ⊡ SC^20^/FM^Ø^ structures with Ø < 5 μm, nor for ⊙ SC^20^/FM^Ø^ structures with Ø < 2 μm, yet the SC stray magnetic field generated by Ø = 20 μm structures should allow the magnetic imprint (see Supporting Information, Section 4). Indeed, micromagnetic modeling show that the stray field originating from ⊙ SC^20^ structures could allow the imprint of magnetic radial vortices in Py down to a lateral size of 900 nm (Supporting Information, Section 3).

We attribute this behavior to the presence of surface defects (CuO precipitates).^16^ The presence of CuO precipitates resulting from the growth of the YBCO (Figure 1a–d) avoids the formation of a continuous film of the ferromagnetic Py. The film grown on top of the precipitates is effectively disconnected from the Py at the surface of YBCO due to their large distances and low total moment. The presence of nonmagnetic regions within the FM layer has an effect in the pinning and stabilization of magnetic domains and spin textures. The pinning of the domain walls can lead to a reduction of the domain wall energy (local energy minimum) and to a local increase of the coercivity and saturation fields^41^ (Supporting Information, Section 4). This is not surprising, since previous studies in submicrometer geometries confirmed that defects like polycrystallinity and geometrical confinement, enhance pinning and stabilization of complex topologically nontrivial textures like vortices and skyrmion tubes.^42^ Consequently, the effective anisotropy field increases, and larger magnetic fields are necessary to erase the initial spontaneous domain structure. The relative importance of surface defects increases as the size of the Py element is reduced, and so, the anisotropy field eventually surpasses the available SC stray field, preventing the imprint of smaller radial vortices.

Strategies to overcome the increase in coercive field due to the presence of defects would require the elimination of defects, when possible, or increasing of the SC stray field. The later could be achieved by increasing the critical current of the SC. It is worth to mention that in this case, the gain in size reduction would still be limited by the size and spatial distribution of defects as well as hindered by the tendency of the magnetic ground state to evolve toward a normal vortex state.^37^

**Figure 5:**
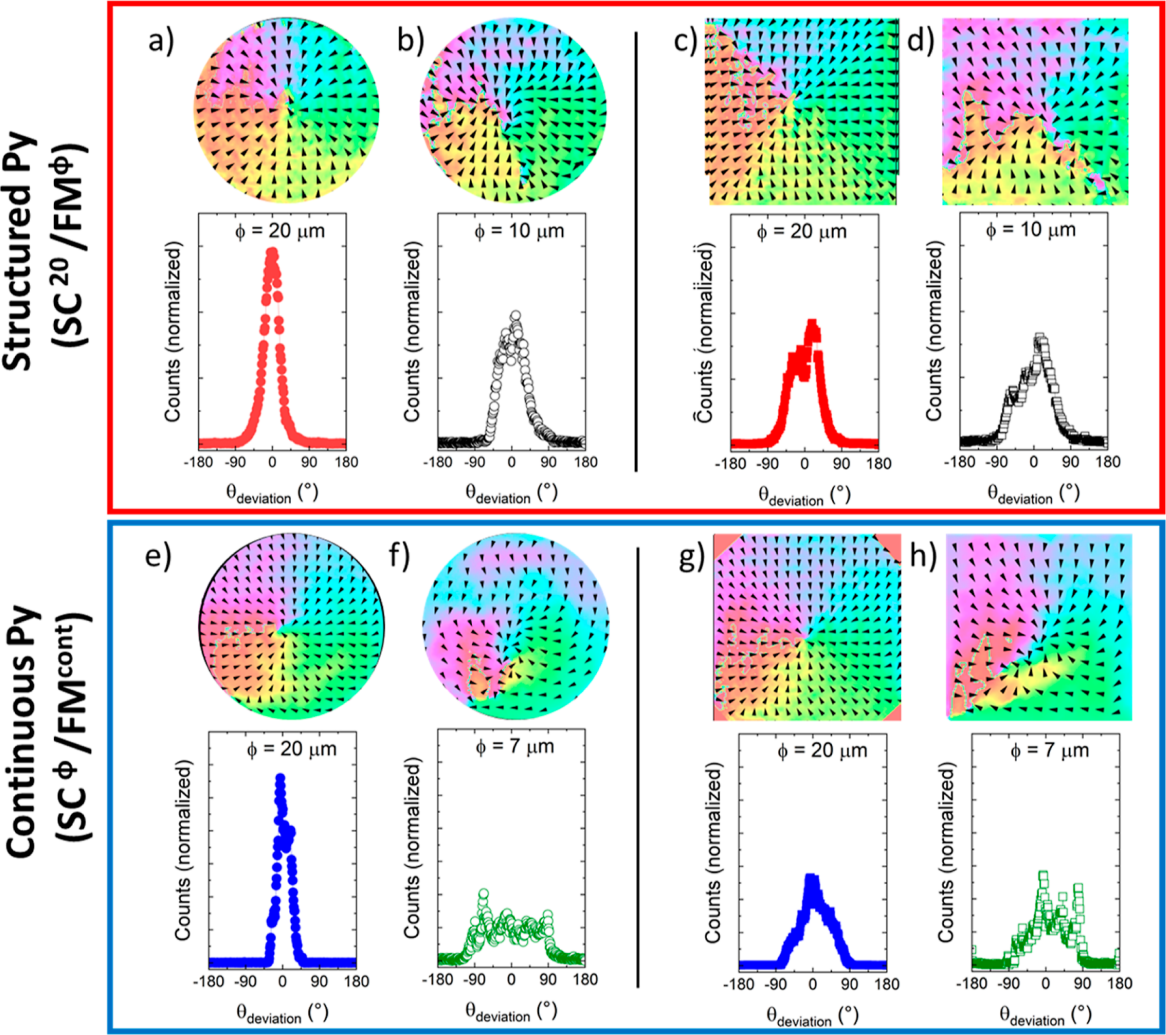
Space resolved maps of the magnetization direction orientation (arrows) at 140 K (*T* > *T*_C_) after the SC imprint of a radial vortex at 50 K (*T* < *T*_C_) averaged over 10 similar structures (see Methods), and histograms of the angular deviation at 140 K, see main text for its definition. Top: structured Py structures. (a) ⊙ SC^20^/FM^20^, (b) ⊙ SC^20^/FM^10^, (c) ⊡ SC^20^/FM^20^, and (d) ⊡ SC^20^/FM^10^. Bottom: continuous Py structures. (e) ⊙ SC^20^/FM^cont^, (f) ⊙ SC^10^/FM^cont^, (g) ⊡ SC^20^/FM^cont^, and (h) ⊡ SC^10^/FM^cont^.

For those structures for which the SC stray field surpasses the anisotropy field, the presence of surface defects can have a beneficial impact on the stabilization of the imprinted swirling domains across a wide temperature range. Increasing the temperature above the *T*_C_ leads to the disappearance of the SC stray field (which stabilizes the imprint). For defect-free samples, this leads to the magnetic relaxation of the system toward a normal vortex state (Supporting Information, Section 3) which core polarization is determined by the z-component of the SC stray field. The calculated coercive field and saturation magnetization of the Py with defects can be used to estimate the energy barrier that needs to be overcome to relax the imprint, in 8000 J/m^3^, which is comparable to the anisotropy energy of the Py, 3500 J/m^3^. Conversely, the presence of surface defects can partially stabilize the imprint (Supporting Information, Section 6). Indeed, the temperature-dependent XMCD data shown in Figure 4 reveal that despite some relaxation, individual structures partially retain above *T*_C_ a memory of the radial vortex imprinted state. This is evidenced in Figure 5 where we plot space-resolved maps of the magnetization direction on SC/FM hybrids of different type (continuous or structured Py), shape (square or circle), and lateral size, at *T* > *T*_C_ (140 K) after SC imprint at *T* < *T*_C_ (50 K) (see Methods for details). The angular distribution of the magnetization direction for the largest structures (Ø = 20 μm), independently on whether they are squares or discs or whether they are made of structured (Figure 5a,c) or continuous Py (Figure 5b,d), resembles that of a radial vortex despite no SC stray-field present. Decreasing the size of the imprint leads to a partial departure from the radial vortex state, more severe in the case of samples made of continuous Py. The later, suggests that the dipolar fields of Py domains (in the relaxed state) compete with the imprint to reduce retention, i.e., the formation of (large) domains at the boundary between the imprinted region in continuous samples is a driving force for lower imprint and weakened retention compared to structured samples (Supporting Information, Section 5).

Further details concerning the particularities of the SC imprint and memory effect for ⊡ and ⊙ structures can be obtained by comparing the magnetic domain state experimentally observed at 140 K with that expected for an ideal radial vortex magnetic distribution. This comparative analysis is performed by the introduction of an angular deviation parameter (θ_deviation_). This parameter quantifies, at each position across the structure, the local angular variance between the magnetization direction at 140 K, as depicted by the arrows in Figure 5, and the expected orientation for a radial vortex. Histograms for θ_deviation_ are included in Figure 5 for each of the structures for which the space resolved maps of the magnetization direction has been obtained at 140 K. Disc-shaped structures of Ø = 20 μm, showing the largest retention of the imprint, show the narrowest distribution of θ_deviation_ with full width at half-maximum (fwhm) of ±20°. Decreasing the size of the structures and/or imprinting squares instead of discs leads to a flattening and a broadening of the angular dispersion of θ_deviation_, more prominent for nonstructured samples.

Particularly relevant is the fact that the fwhm of Ø = 20 μm square structures (Figure 5c,g) is approximately twice as wide as the one corresponding to discs of identical lateral size. These differences can be attributed to the presence of magnetic domain walls along the diagonals in the square structures after the low temperature imprint. These domain walls, separating head-to-head magnetic domains, increase the dipolar energy. At *T* > *T*_C_, in the absence of the stabilizing SC stray field, the magnetostatic energy is minimized in the vicinity of those domains by relaxing the imprinted magnetic domain state. Regions away from the diagonals tend to be more stable. In comparison, the symmetry of the discs leads to a magnetic domain imprint with no DWs as the radial magnetization rotates continuously around the center, with no preferred magnetization orientation. Micromagnetic modeling confirms that the absence of DWs within the discs leads to an easier impression and to a higher stability of the imprinted state as compared to similar size square-like structures (Figure 5b and Supporting Information, Figure S11).

We finally note the asymmetry around 0° in the distribution of θ_deviation_ for square elements corresponding to samples with structured Py. This asymmetry is present despite the absence of FM regions between structures. We tentatively ascribe its origin to the presence of some residual field within our experimental setup. Relevant enough, disc structures are apparently more robust against this perturbation as observed by the higher symmetry of the θ_deviation_ deviation distribution for disc structures of Ø = 10 μm (Figure 5b) as compared to Ø = 20 μm and Ø = 10 μm square-shaped elements (Figure 5c,d).

## Conclusions

Radial vortex magnetic domain configurations have been crafted in Py by means of the stray field generated by SC structures down to a 2 μm lateral size. This minimum size threshold is reached for SC/FM hybrid systems were structured Py is deposited on top of the largest superconducting elements studied. This configuration minimizes dipolar interactions and maximizes the stray field of the superconductor. Structures with a lateral size of 1 μm could not be imprinted despite micromagnetic simulations for defect-free Py showing its feasibility. It is concluded that the presence of surface defects increases the coercive and saturation fields to values above those generated by the SC structure, thus hindering the imprint.

Hybrid SC/FM structures retain memory to some extent of the imprinted magnetic domain state at *T* > *T*_C_ despite the disappearance of the stabilizing SC stray field. Micromagnetic modeling indicates that instead, the stability above the superconducting transition temperature is provided by microstructural defects. In the absence of defects, the system would relax to a conventional magnetic vortex state with its polarity determined by the z-component of the stray-field of the superconductor.

Overall, disc-shaped structures with structured Py provide an enhanced preservation of the imprint compared to square geometries due to confinement and circular symmetry, preventing the formation of head-to-head 90° magnetic domains otherwise energy-penalizing.

Future work will be directed to optimize the introduction of defects (size, shape, number, etc.) within the FM structures to improve the stabilization of the magnetic structures imprinted by the SC stray fields. Hybrid SC/FM heterostructures open an appealing direction for SC-field design and manipulation of magnetic textures in soft magnetic materials with great potential to shape spintronic applications based on magnetic textures. Spin textures with various spin arrangements (skyrmions, merons, and hopfions) are being considered as the basis for spin-based memory devices. Yet, a major drawback limiting their large-scale integration is the energy efficiency and size requirements of current paths. Our research has uncovered a new strategy for energy efficient writing of memory elements exploiting the superconducting flux, which may signal an interesting new avenue in future spin-based superconducting electronics.

## Methods

Sample fabrication: electron beam lithography was performed in a Raith50 module mounted on a Zeiss EVO 50 scanning electron microscope to obtain square and disc patterns with different sizes. The first step was performed in a YBCO single layer using a negative resist to cover parts of the layer, which was later chemically etched. A second lithography step was performed to define square and disc holes on top of the YBCO square and holes using a positive resist. Py was grown on top of the sample, and then, a lift-off was performed to eliminate Py outside of devices.

PEEM imaging: X-ray PEEM is a magnetic and element selective technique with a resolution of about 30 nm. Unlike many other techniques (e.g., magnetic force microscopy), X-ray PEEM delivers direct information about the magnetization, and the element selectivity guarantees that the recorded magnetic information comes only from the element under investigation. This is important, as other techniques would also prove the magnetic field generated by the superconductor. Magnetic sensitivity arises from the difference in absorption of circularly polarized radiation with left and right helicity from a magnetic element.^43^

Experiments were done at the PEEM station at the UE49/PGMa beamline of the synchrotron radiation source BESSY II of the Helmholtz-Zentrum Berlin.^44^ The angle of incidence of the incoming radiation with respect to the sample surface was 16°, which ensured a sizable projection of the in-plane magnetization of the Py layer along the beam propagation direction, which gives rise to the XMCD signal.

Magnetic imaging was always performed in zero external field after an out-of-plane magnetic field pulse. Positive polarity corresponds to fields outside the page plane. The maximum pulse amplitude was ± 100 mT with a pulse duration of 0.5–1.0 s and increasing/decreasing field rates of 10 mT/s. XMCD Images were collected at the Fe *L*_3_-edge (707 eV) for incoming circularly polarized radiation with right (σ^+^) and left (σ^−^) helicity, respectively. A total of 30 images, each with a 3 s integration time, were collected per helicity. Each image was normalized to a bright field image and drift corrected before their averaging. The XMCD images were obtained as (σ^–^ – σ^+^)/(σ^–^ + σ^+^), where σ^+^ and σ^–^ were the averaged images for right and left circularly polarized radiation, respectively.

2D maps of the magnetization direction were computed from two XMCD images obtained at 0° and 90° azimuthal rotation of the sample. XMCD images at 0° and 90° were averaged over ten similar structures.

### Calculation of the Stray Field of the SC Structures

The stray magnetic field of disc- and square-shaped superconductor structures at the top surface of SC elements was calculated by using magnetostatic finite-element method simulations, assuming a current density equal to *J*_c_ in the superconducting volume.

### Micromagnetic Simulations

Micromagnetic simulations by means of Mumax3^45−47^ have been performed to investigate the role of defects in the stabilization of magnetic radial vortices imprinted in Py as the temperature is raised above the superconducting transition temperature of YBCO. The ferromagnetic domain state below and above the superconducting transition temperature of YBCO was simulated by considering the presence or absence of a superconductor magnetic stray field, respectively. Ferromagnetic Py disc- and squared-shaped structures with dimension alike as those reported within the paper were simulated with the effective parameters of the Py layer in proximity with YBCO, calculated in previous works^48^ (*M*_sat_ = 0.86 MA/m; *A*_ex_ = 13 pJ/m, α = 3.9 × 10^–3^). The presence of defects was imitated by the random inclusion of 400 holes (420 nm diameter) over an area of 20 × 20 μm.

## Supporting Information Available

Stray field generated by the superconductor structures, differences in XMCD imaging of radial- and Landau-magnetic distributions, relaxed vortex state for defect-free hybrid SC/FM disc-shaped structures after removal of the SC stray field, influence of surface defects for small structures, effect of dipolar field arising from ferromagnetic regions in between structures on the *T* > *T*_C_ magnetic state after low-temperature SC imprint, and memory effect of imprinted magnetic radial vortices for hybrid SC/FM structures with defects (PDF)
