# Supplementary material for: Size-Dependence and High Temperature Stability of Radial Vortex Magnetic Textures Imprinted by Superconductor Stray Fields
Source: ACS Appl Mater Interfaces. 2024 Apr 2;16(15):19681–90. doi: 10.1021/acsami.3c17671 (PMC11040579; doi:10.1021/acsami.3c17671)
Supplement: Supplementary file 1 — am3c17671_si_001.pdf [file am3c17671_si_001.pdf]

Supporting Information

Size-dependence and high temperature stability of radial vortex magnetic textures imprinted by superconductor stray fields.

David Sanchez-Manzano[1,2], Gloria Orfila[2], Anke Sander[1], Lourdes Marcano[3,4], Fernando Gallego[2], Mohamad A. Mawass[3,‡], Francesco Grilli[5], Ashima Arora[3], Andrea Peralta[2], Fabian A. Cuellar[2], Jose A. Fernandez-Roldan[6], Nicolas Reyren[1], Florian Kronast[3], Carlos Leon[2], Alberto Rivera-Calzada[2], Javier E. Villegas[1], Jacobo Santamaria[2], Sergio Valencia[3,*].

[1] Laboratoire Albert Fert, CNRS, Thales, Université Paris-Saclay, 91767 Palaiseau, France.

[2] GFMC. Dept. Física de Materiales. Facultad de Física. Universidad Complutense. 28040 Madrid, Spain.

[3]Helmholtz-Zentrum Berlin, Albert-Einstein Str. 15, 12489 Berlin, Germany.

[4]Dept. of Physics, Faculty of Science, University of Oviedo 33007 Oviedo, Spain.

[5]Karlsruher Institut für Technologie, Institut für Technische Physik, 76344 Eggenstein-Leopoldshafen, Germany.

[6]Helmholtz-Zentrum Dresden-Rossendorf e.V., Institute of Ion Beam Physics and Materials Research, 01328 Dresden, Germany;

‡Present address: Department of Interface Science, Fritz Haber Institute of the Max Planck Society, 14195 Berlin, Germany.

*Corresponding author: sergio.valencia@helmholtz-berlin.de

1.- Stray field generated by the superconductor structures: lateral size dependence

The x, y and z components of the magnetic field generated by 250 nm thick YBCO superconductor squares after a magnetic field pulse of +100 mT has been computed as function of their lateral size (Ø), see methods for details. The superconducting current present within the YBCO structure after the magnetic field pulse leads to a space-dependent magnetic stray field generated by the SC which strength and direction varies from point to point, see Figures S1 and S2 where the z and x components of this field are depicted for Ø = 20, 10, 5, 2, and 1 μm.

Devices for which the Py has been structured have been grown on top of YBCO structures with a lateral dimension of 20 μm. Figure S3 depicts x, y and z components of the magnetic stray field generated by a superconducting disc- and squared-shaped (⊙, ⊡)$SC^{Ø}$.

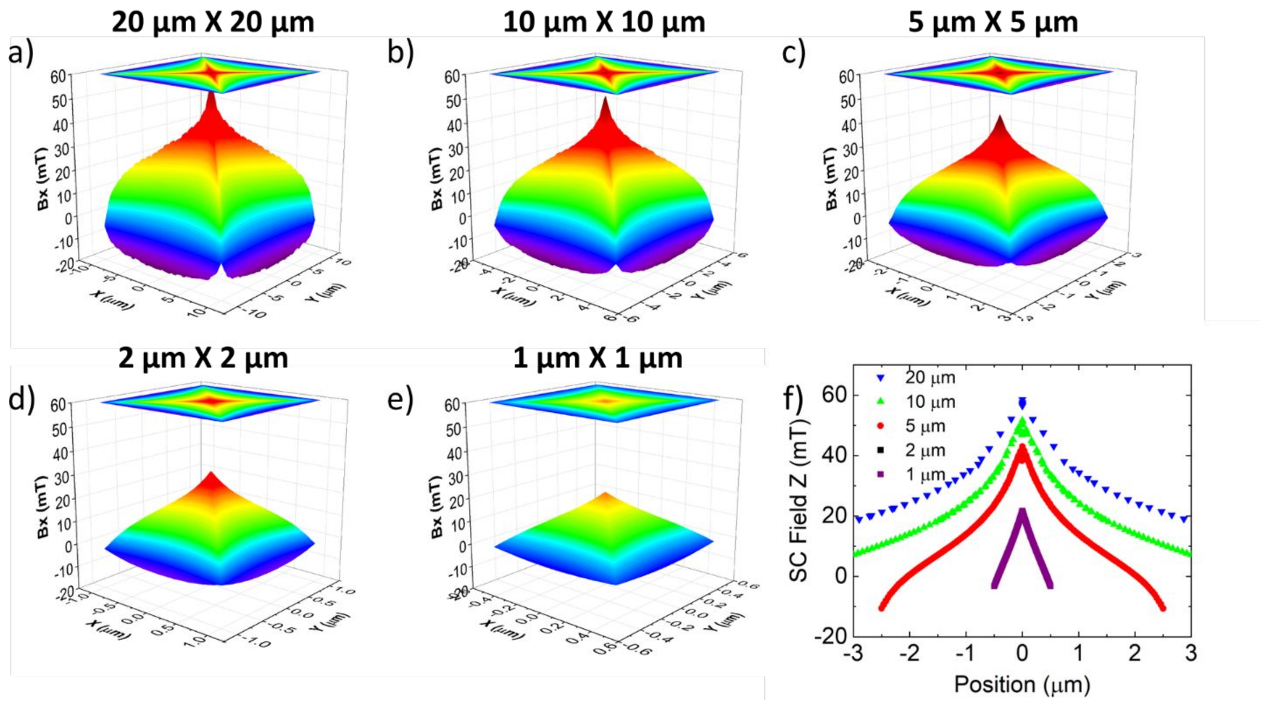

Figure S1 a)-e) Z component of the strain field generated by a squared 250 nm thick SC structure after an out-of-plane magnetic field pulse of +100 mT. Lateral dimensions indicated on top of each panel. f) Profiles of the Z-component of the magnetic stray field of the SC measured from a straight line orthogonal to the edges and crossing the center. The reduction of the lateral size leads to an overall reduction of the magnetic stray field.

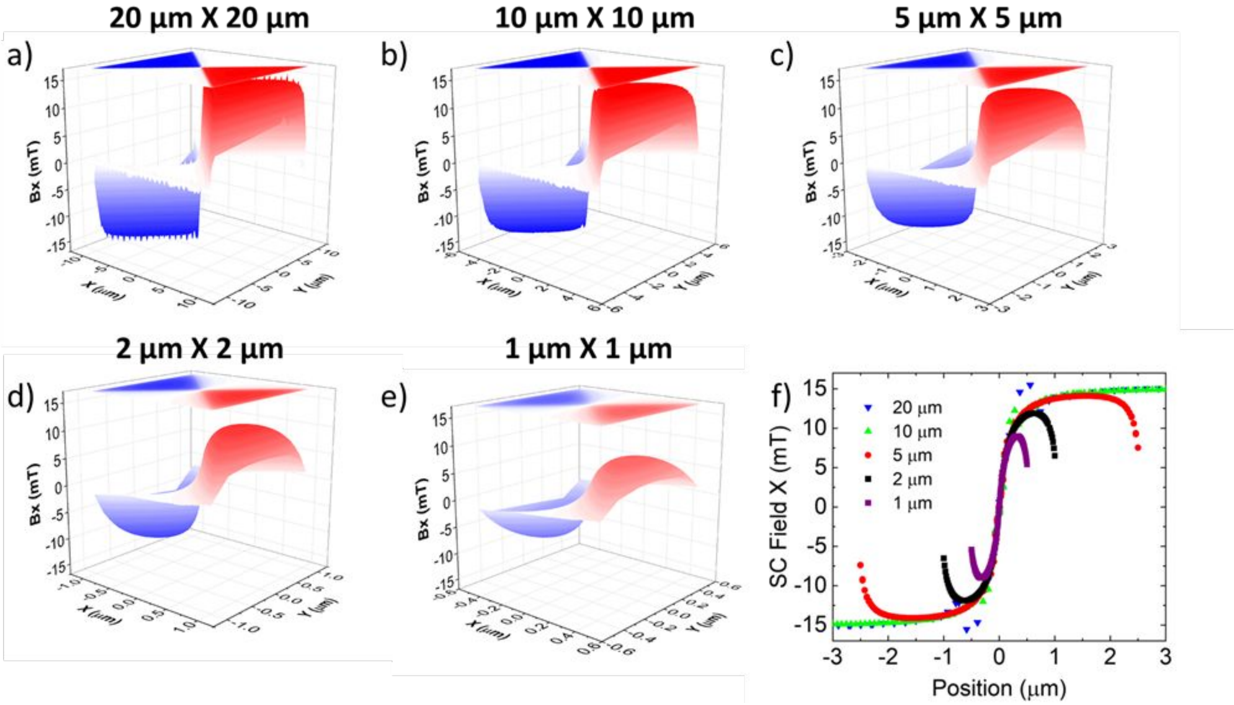

Figure S2. a)-e) X component of the SC stray-field for 250 nm thick YBCO square structures with lateral dimensions as indicated after an out-of-plane field pulse +100 mT. f) Profiles of the x-component of the magnetic stray field of the SC measured from a straight line orthogonal to the edges and crossing the center. The reduction of the lateral size leads to an overall reduction of the magnetic stray field. The y component shows the same behavior as the x one.

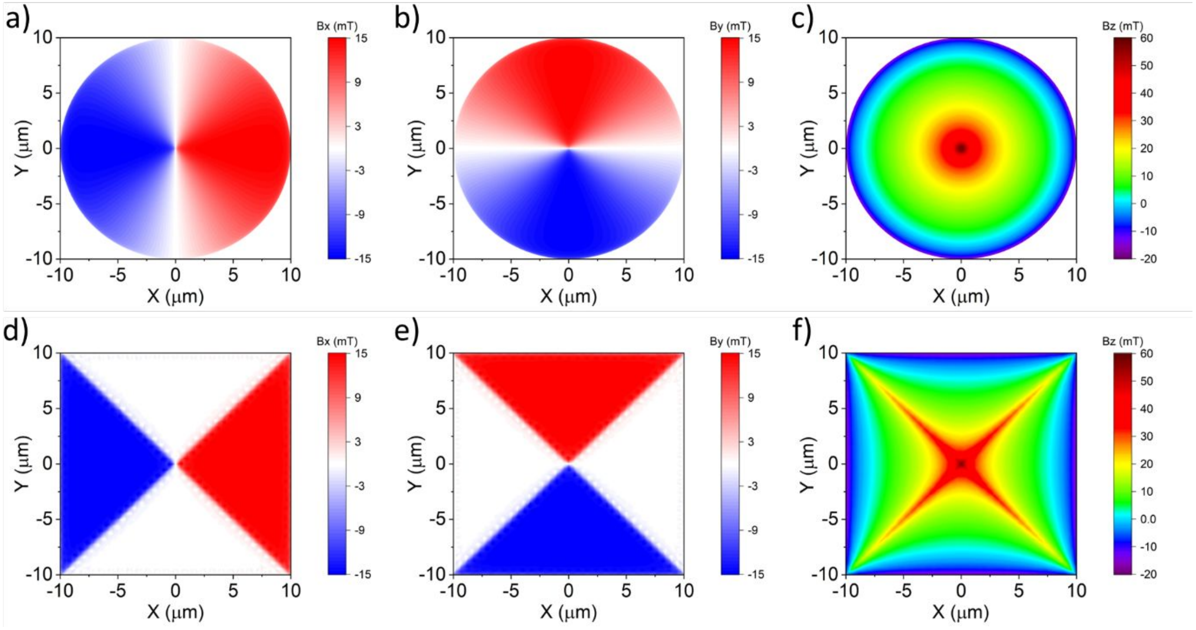

Figure S3. 2D representation of the X, y and Z components of the SC self-field (in Gauss) for a 20 µm size and 250 nm thick YBCO circular (a-c) and square (d-f) elements after an out-of-plane field pulse +100 mT.

2.- Differences in XMCD imaging of radial- and Landau- magnetic distribution.

In Landau vortex states, the magnetization curls (clockwise or anticlockwise) around the center of the structure to minimize the magnetostatic energy. In a radial vortex the magnetization direction points towards or away from the core on a radial direction orthogonal to the contour of the SC structure.

XMCD is proportional to the projection of the magnetization along the x-ray beam propagation direction. The XMCD signal is maximized for a magnetization direction parallel to the beam propagation direction.

Figure S4 sketches the expected XMCD images for radial and normal magnetic vortices measured with the incoming x-ray beam, along the blue arrow. Both, radial- and normal-vortex states show similar XMCD images but rotated by 90°.

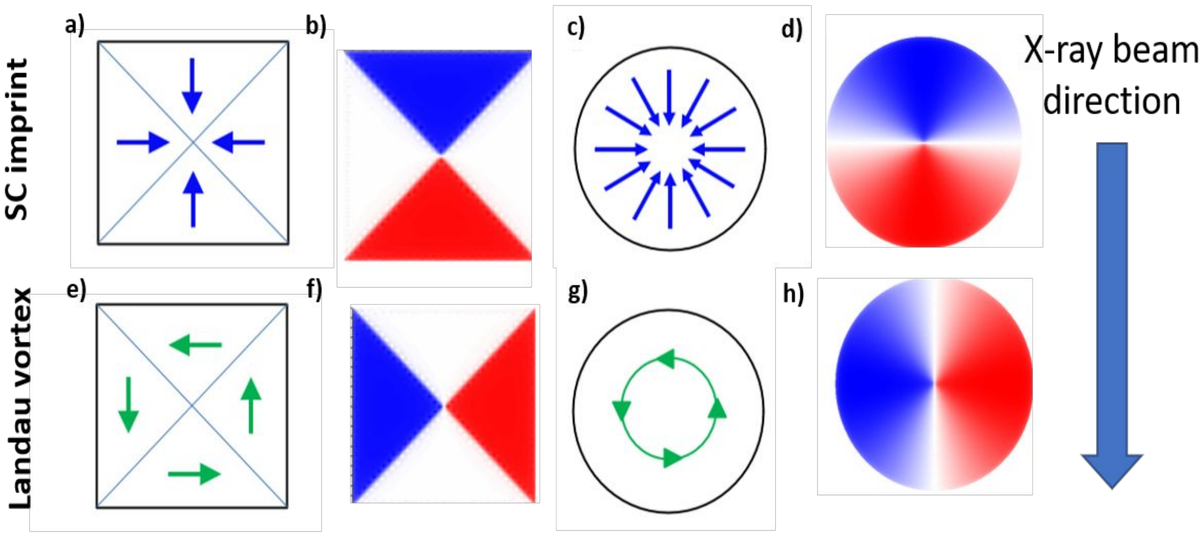

Figure S4. Comparison of the magnetization orientation distribution for radial vortex (panels a and c) and Landau or vortex-like magnetic domain distributions (panels e and h) for square and disc-shaped domains. Panels b and d and panels f and g simulated the expected XMCD images taking into account the experimental geometry, i.e. the orientation of the X-ray beam with respect to the structures.

3.- Relaxed vortex state for defect-free hybrid SC/FM disc-shaped structures after removal of the SC stray field.

We carried out finite difference micromagnetic modelling for hybrid ⊙$SC^{20}/FM^{\o}$ structures with no defects as function of the damping factor of Py to determine its magnetic domain state in the presence of the SC stray field and once this has been removed. Simulations have been performed for SC fields resulting

from both +100 mT and -100 mT field pulses (see Figure S3) with a customized version of Mumax 3[1–3] and postprocessing.

A prominent example of the switching process is depicted in figure S5, showing states before (Figure S5a), during (Figure S5b) and after (Figure S5c) the application of the SC field for a 3 µm diameter Py structure. Figure 5b evidences a radial distribution of the magnetization direction with a string distortion near the vortex core and at the disc boundaries. The distortion shapes the core in an spiral-like flavor in agreement to experimental XMCD images in Figure 4 Radial vortex-like states can be imprinted down to a lateral size of 900 nm (Figure S6).

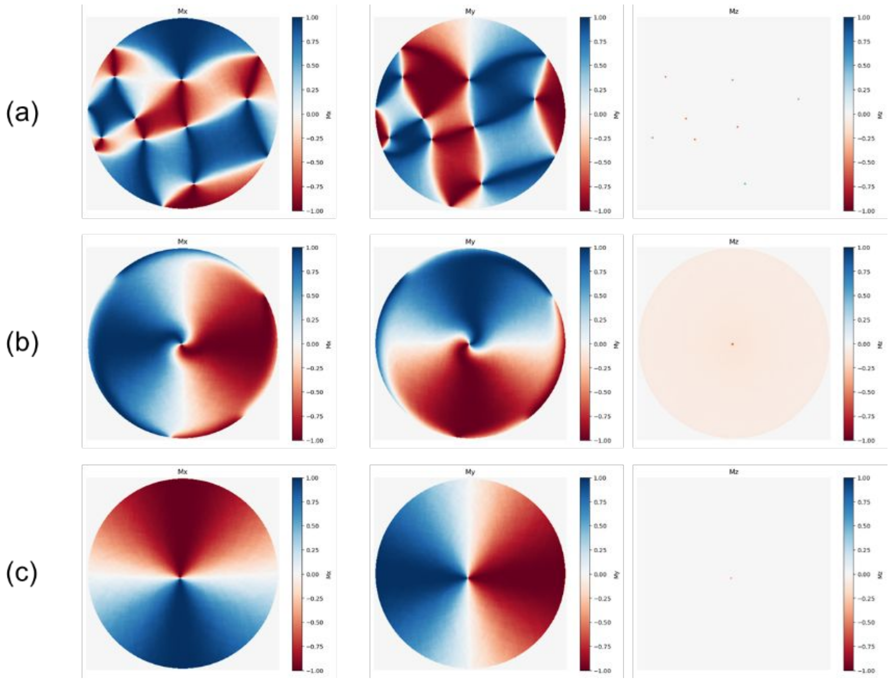

Figure S5 Examples of selected magnetic states before the application of the SC field (a), during the SC field (b) and after removing the SC field (c) *in a Py disc with diameter of 3 µm. Each row shows the components x, y, z of the magnetization. The z- axis is perpendicular* to the disc plane.

For all disc diameters investigated the suppression of the SC stray field leads to a normal vortex state where the magnetization curls around the center. Figure S7 summarizes the results from the simulations. The helicity of the resulting vortex state is independent of the damping factor and suggest that the helicity is likely to be determined by the microstructure. On the other hand, the polarity of the vortices is in most of the cases determined by the z-component (positive or negative) of the SC stray field (Figure S7a,b).

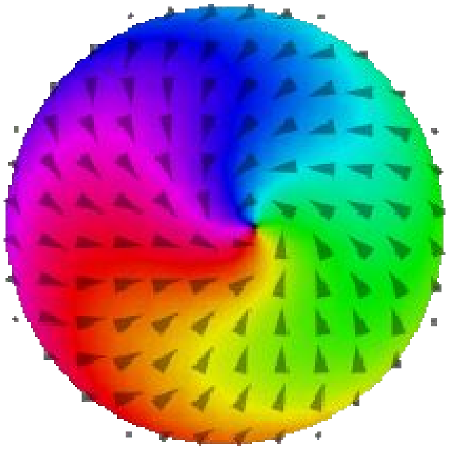

Figure S6 2D map of the magnetization orientation from micromagnetic simulations for defect-free Py ⊙ structure of 900 nm diameter on top of a *SC*[20] structure. Magnetic state in the presence of a SC resulting from a magnetic field pulse of +100 mT. The SC field allows the imprint of a radial vortex. Arrows indicate the local magnetization direction.

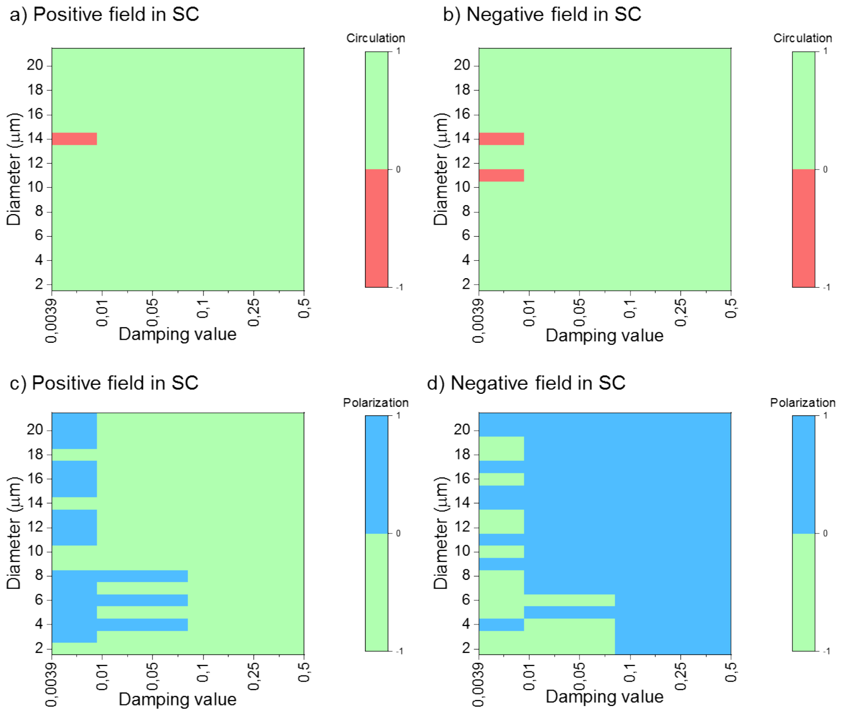

Figure S7 Diagrams of the circulation and polarity of a vortex state in a disc with diameters between 1 and 20 µm after the application of either positive (a, c) or negative (b, d) currents in the superconductor. Values have been obtained solving Landau-Lifshitz-gilbert Equation with the SC field. The switching process is high dependent on the damping value close to realistic values of materials.

4.- Influence of surface defects for small structures.

To address the role played by surface defects we have simulated in-plane magnetic hysteresis loops, by means of Mumax3 micromagnetic simulations, for a Py disc-shaped structure with diameter ϕ = 1, 2 and 5 µm without (Figure S8a) and without (Figure S8b) surface defects (see methods). While the extraction of quantitative information needs to be handled with care[1], a qualitative comparison between the results obtained for the different cases is feasible. Simulations corresponding to the defect-free sample show in symmetric loops with relatively low coercive (H_c) and saturation fields (H_s), exception of the ϕ = 1 µm structure owing to its magnetic ground state being a vortex. Besides the expected increase in H_c as the Py island size is reduced (panel a), it is observed that the introduction of defects leads to a decrease of the remanent magnetization for all structures and to an increase of both H_c and H_s (panels c-e) for ϕ ≥ 2 µm.

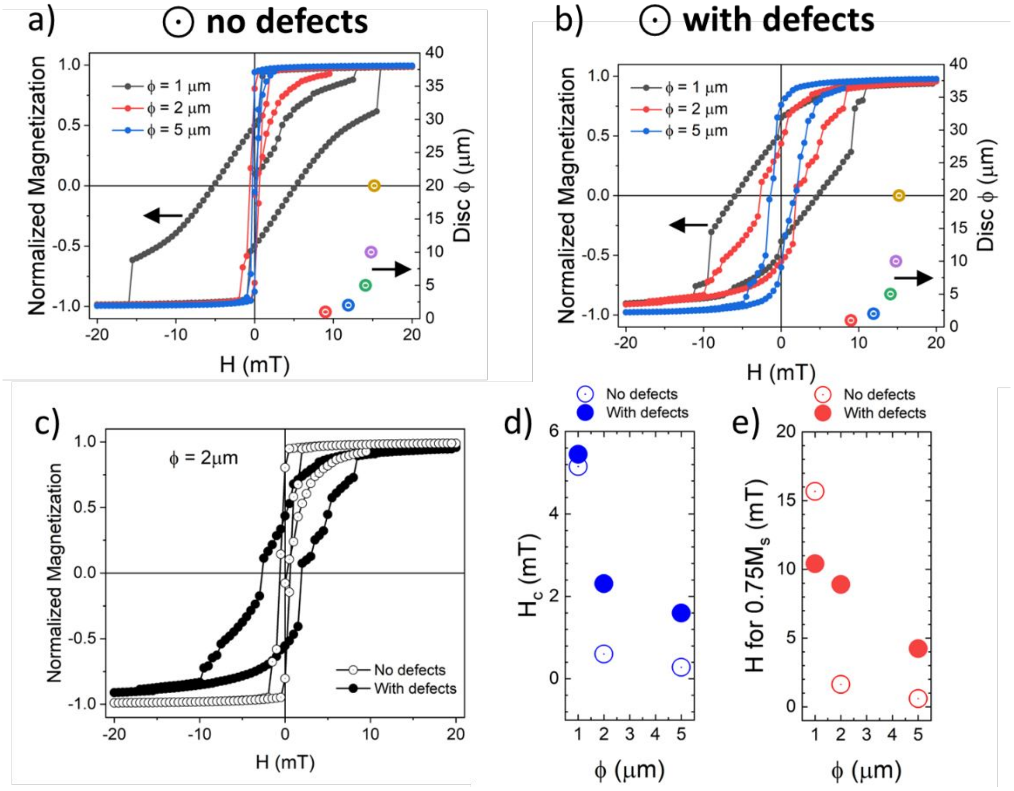

Figure S8 Micromagnetic simulations for 4 nm thick Py disc structures with diameter Ø = 1, 2 and 5 µm. a) Hysteresis loop between ± 20 mT for FM structures with no defects (colored dots show the strength of the in-plane component of the SC stray field as function of SC island diameter (right axis). b) Hysteresis loop between ± 20 mT for FM structures with defects.

The presence of defects leads to asymmetric loops with magnetization jumps associated to the nucleation and depinning of magnetic domains at defect positions. As a consequence, the coercive field for ϕ ≥ 2 µm increases by a factor 3 to 5 as compared to the defect-free cases. Similarly, the saturation field,

typically below 5 mT increases to values beyond the range used for the simulations (±20 mT). It is worth to remark that the size of the microstructures is large enough for the spontaneous formation of domain walls.

It is crucial to note that a direct quantitative comparison between the results of the simulations and the experimental data is not possible as the simulations do not capture the "reality" of the samples (exact dimensions and granularity, roughness, temperature…). Moreover, the loops are simulated assuming a field applied along a specific direction whereas the imprint is done by applying a space dependent magnetic field profile generated by the SC island. This disparity is evident in the hysteresis loops obtained for the smallest structure. For instance, a disc of ϕ = 1 µm without defects exhibits an H_c which is significantly larger than that obtained for ϕ = 2 µm, owing to its magnetic ground state being a vortex. Although the saturation field within the simulations (Hs ~ 18 mT) exceeds the in-plane component of the SC island´s stray field (~ 15 mT), micromagnetic simulations for a 900 nm disc show that the imprint is possible (see, Figure S6).

Taking this into account, we can conclude from the simulations that the presence of defects, pining magnetic domain walls, does have an impact increasing the local H_c and H_s. This increase competes against the stray field generated by the SC island difficulting or even precluding the imprint.

5- Effect of dipolar field arising from ferromagnetic regions in between structures on the T > Tc magnetic state after low temperature SC imprint.

In Figure S9 we show the impact of the dipolar field originating from ferromagnet regions in between structures on the resultant magnetic state at T > Tc after disappearance of the SC magnetic stray field. Panel a and b show polar histograms of the angular orientation of the magnetization at T = 140 K for ⊡ SC^{20}/FM^{cont} and ⊡ SC^{20}/FM^{20} structures after low temperature imprint. Histograms have been obtained on domains with an original imprint direction of the magnetization at T= 50 K of 0° (red), 90° (yellow), 180° (green) and 270° (blue). Structures with a continuous Py layer (panel a) show at 140 K a clear tendency to align along directions imposed by ferromagnetic regions around SC structures (black curve). Samples with structured Py (panel b) show more pronounced retention of the original imprinted state albeit some deviation towards a seemingly common direction. The source of this preferred relaxation

direction remains unknown, but it is likely associated with some residual field present in the experimental setup.

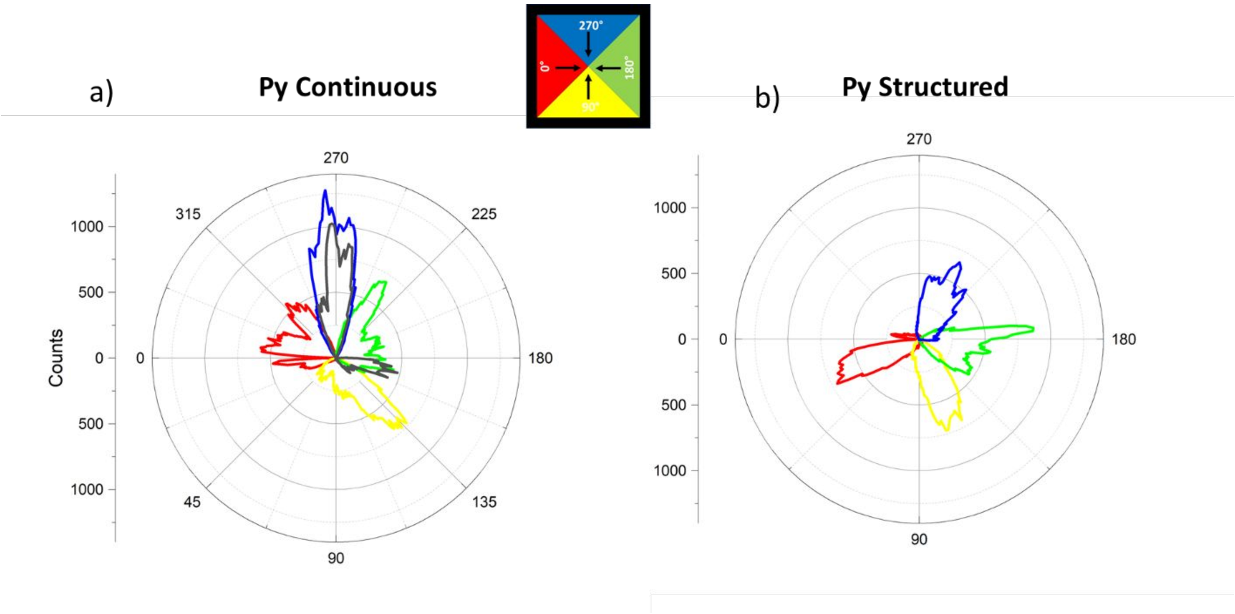

Figure S9 Polar plots for SC/FM hybrid square structures of 20 µm lateral size for the cases of a) continuous Py layer and b) structured Py on top of SC elements. Histograms have been obtained from regions where the original low temperature imprint sets the magnetization along 0°, 90°, 180° and 270° directions (see sketch on top of histograms)

6.- Memory effect of imprinted magnetic radial vortices for hybrid SC/FM structures with defects.

Micromagnetic simulation by means of Mumax3[1–3] have been performed for a total of 10 (⊡,⊙)SC[20]/FM[20] structures to determine the magnetic domain state after removal of the SC stray field. Each simulated structure contained 400 non-magnetic defects (diameter of 420 nm) aleatory distributed.

Figure S10 depict 2D magnetization maps for single ⊡ and ⊙ structures with the SC stray field “on”. The magnetic domain state resembles the radial vortex magnetic state obtained for defect-free structures (Figure S5). Particularities of the magnetic state depend on the specific defect distribution. Panels c) and d) of Figure S10 shows the resulting magnetic domain state after the removal of the SC stray field. These images have been obtained by averaging all 10 similar structures, as done for the XMCD in the main manuscript, in order to determine their common behavior. Note that despite the disappearance of the SC stray field, which stabilizes the imprint, the resulting magnetic domain state resembles the radial vortex state imprinted by the superconductor. This is in contrast with what it is observed for defect-free structures for which the removal of the SC stray field leads to the system to relax to a vortex state.

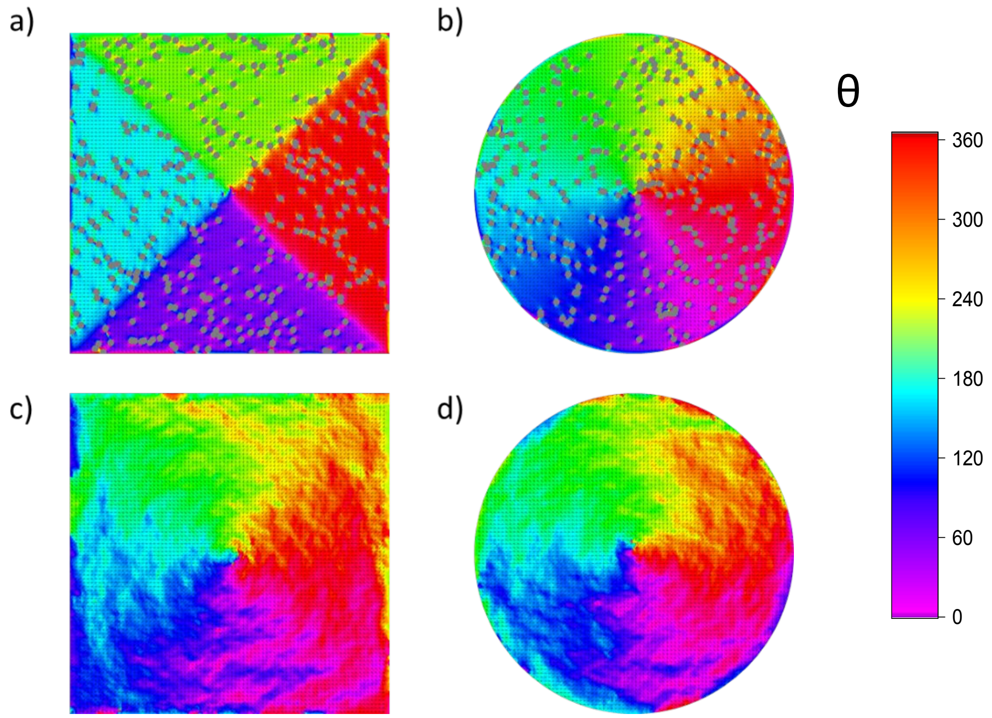

Figure S10 2D maps of the magnetization direction obtained from micromagnetic simulations for **(⊡ ,⊙)***SC*^{20}*/FM*^{20} structures with defects. a) and b) In the presence of the SC stray field the magnetic domain structure resembles that of a radial vortex c) and d) Magnetization maps resulting from the removal of the SC stray field averaged over 10 similar structures with aleatory defects distribution. The resulting magnetic domain distribution resembles that depicted in a) and b). That is, there is no relaxation to a vortex state as in the case of defect-free structures (**Figure S5**).

Figure S11a,b shows 2D maps of the resulting magnetization direction (arrows) as well as of the deviation angle as compared to an ideal radial vortex. Panels c and d show the corresponding histograms. Results agree qualitatively with the experimental observations, thus highlighting the role of defects on the stabilization of the SC imprinted patter at T > Tc where the SC stray field is suppressed. Note larger deviation angles close to the diagonals of the square, positions where a domain wall is imprinted by the SC.

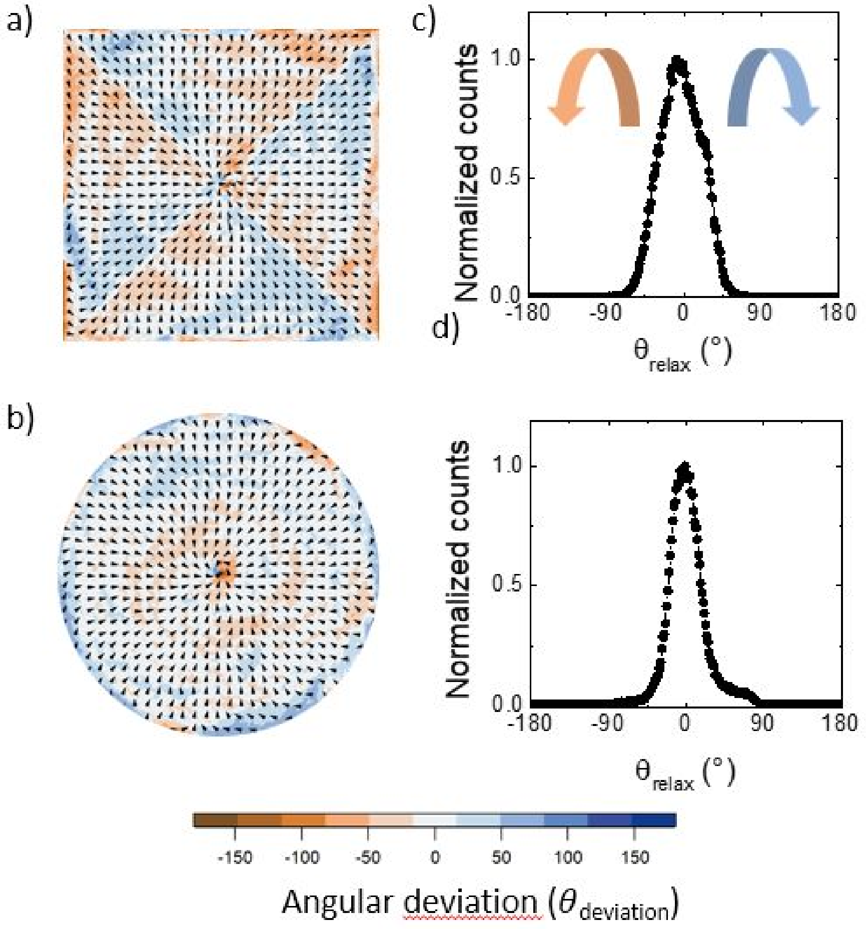

Figure S11 Micromagnetic simulations: a) and b) Arrows: Angular orientation of the magnetization after suppression of the SC stray field after the SC imprint of a radial vortex magnetic domain state averaged over 10 similar **(** ⊡ ,⊙**)**SC^{20}/FM^{20} structures (see methods) in order to highlight their common behavior. Color: Angular deviation between the angular orientation at 140 K and that expected for an ideal radial vortex. c) and d) histograms of the angular deviation represented in panels a) and b). Color arrows in panels c) indicate the direction of the angular deviation.

Bibliography

(1) Vansteenkiste, A.; Leliaert, J.; Dvornik, M.; Helsen, M.; Garcia-Sanchez, F.; Van Waeyenberge, B. The Design and Verification of MuMax3. *AIP Adv.* **2014**, *4* (10), 107133.

(2) Leliaert, J.; Van De Wiele, B.; Vansteenkiste, A.; Laurson, L.; Durin, G.; Dupré, L.; Van Waeyenberge, B. Current-Driven Domain Wall Mobility in Polycrystalline Permalloy Nanowires: A Numerical Study. *J. Appl. Phys.* **2014**, *115* (23), 233903.

(3) Exl, L.; Bance, S.; Reichel, F.; Schrefl, T.; Peter Stimming, H.; Mauser, N. J. LaBonte's Method Revisited: An Effective Steepest Descent Method for Micromagnetic Energy Minimization. *J. Appl. Phys.* **2014**, *115* (17), 17D118.
